# New Appliances and Things Medical

**Published:** 1897-02-20

**Authors:** 


### NEW APPLIANCES AND THINGS MEDICAL.

**[We shall be glad to receive, at our Office, 28 & 29, Southampton Street, Strand, London, W.C., from the manufacturers, specimens of all new preparations and appliances which may be brought out from time to time.]**

### MEAT ESSENCES AND MEAT JUICE.

(Curtis and Co., 48, Baker Street, W.)

The meat essences prepared by the above firm consist of beef, chicken, mutton, veal, and turtle. They appear to be carefully made, and are sent out in large-mouthed glass bottles—a concession to the demands of the medical profession which is made by few manufacturers of these preparations. They are extremely palatable, and can be retained by the most sensitive of stomachs; clinical experience shows that when patients show an aversion to all other kinds of food, these essences or jellies are readily taken. The meat juice by the same firm appears to be a very good sample of expressed juice from red muscle fibre. It is claimed for it that it is made exclusively from English beef. It contains a considerable percentage of unaltered albumin and other meat extractives and salt. The nourishing properties of the meat are therefore afforded to us in this meat juice in the most digestible and assimilable form possible.

### FARINE BREAD.

(Neave and Co., Bickton Mills, Fordingbridge, Salisbury.)

This is an excellent sample of a class of bread which is daily becoming more popular. By a special process of manufacture only the more valuable portions of the wheat berry are utilised in the production of flour from which Farine bread is made. As a food, it has the advantage over ordinary bread in containing less indigestible matter, and in possessing a higher percentage of natural fats. The flavour is excellent, the nutritive value high, and it is easily digested—three of the most important factors that are concerned in the constitution of a first-class bread.

